# Citrate synthase variants improve yield of acetyl-CoA derived 3-hydroxybutyrate in *Escherichia coli*

**DOI:** 10.1186/s12934-024-02444-8

**Published:** 2024-06-12

**Authors:** Hemshikha Rajpurohit, Mark A. Eiteman

**Affiliations:** 1 School of Chemical, Materials and Biomedical Engineering, Athens, GA USA; 2 Department of Microbiology, University of Georgia, Athens, GA 30602 USA

**Keywords:** Batch, Repeated batch, Fermentation, Point mutation, 3-hydroxybutyrate

## Abstract

**Background:**

The microbial chiral product (R)-3-hydroxybutyrate (3-HB) is a gateway to several industrial and medical compounds. Acetyl-CoA is the key precursor for 3-HB, and several native pathways compete with 3-HB production. The principal competing pathway in wild-type *Escherichia coli* for acetyl-CoA is mediated by citrate synthase (coded by *gltA*), which directs over 60% of the acetyl-CoA into the tricarboxylic acid cycle. Eliminating citrate synthase activity (deletion of *gltA*) prevents growth on glucose as the sole carbon source. In this study, an alternative approach is used to generate an increased yield of 3-HB: citrate synthase activity is reduced but not eliminated by targeted substitutions in the chromosomally expressed enzyme.

**Results:**

Five *E. coli* GltA variants were examined for 3-HB production via heterologous overexpression of a thiolase (*phaA*) and NADPH-dependent acetoacetyl-CoA reductase (*phaB*) from *Cupriavidus necator*. In shake flask studies, four variants showed nearly 5-fold greater 3-HB yield compared to the wild-type, although pyruvate accumulated. Overexpression of either native thioesterases TesB or YciA eliminated pyruvate formation, but diverted acetyl-CoA towards acetate formation. Overexpression of pantothenate kinase similarly decreased pyruvate formation but did not improve 3-HB yield. Controlled batch studies at the 1.25 L scale demonstrated that the GltA[A267T] variant produced the greatest 3-HB titer of 4.9 g/L with a yield of 0.17 g/g. In a phosphate-starved repeated batch process, *E. coli ldhA poxB pta-ackA gltA*::*gltA*^[A267T]^ generated 15.9 g/L 3-HB (effective concentration of 21.3 g/L with dilution) with yield of 0.16 g/g from glucose as the sole carbon source.

**Conclusions:**

This study demonstrates that GltA variants offer a means to affect the generation of acetyl-CoA derived products. This approach should benefit a wide range of acetyl-CoA derived biochemical products in *E. coli* and other microbes. Enhancing substrate affinity of the introduced pathway genes like thiolase towards acetyl-CoA will likely further increase the flux towards 3-HB while reducing pyruvate and acetate accumulation.

**Supplementary Information:**

The online version contains supplementary material available at 10.1186/s12934-024-02444-8.

## Introduction

(R)-3-hydroxybutyric acid (3-HB) is a chiral building block for the production of fine chemicals in the chemical, food, and pharmaceutical industry [1, 2], and an advantage of the microbial route for 3-HB synthesis is the ease of generating the pure enantiomer [3]. Microbial synthesis of 3-HB has been accomplished in both bacteria and yeast [1, 3].

**Fig. 1:**
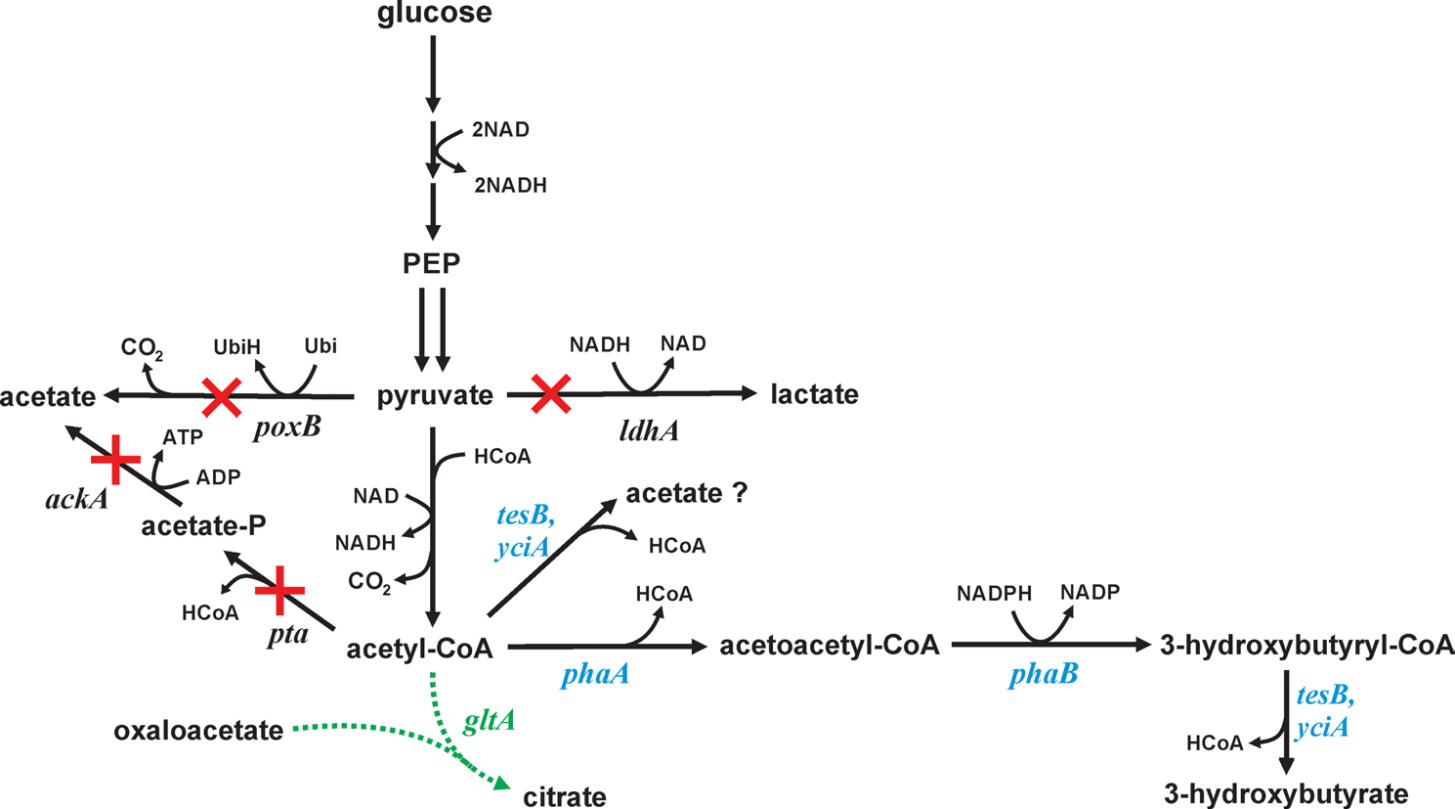
Biochemical pathway to 3-hydroxybutyrate. The *E.coli* strains studied each had deletions in the following genes indicated (**×**): *poxB* (coding pyruvate oxidase), *ldhA* (coding lactate dehydrogenase), *pta* (coding phosphate acetyltransferase) and *ackA* (coding acetate kinase). Several strains additionally had chromosomal mutations in the *gltA* gene, leading to single amino acid substitution citrate synthase variants (green). For 3-hydroxybutyrate production, the *phaA* and *phaB* genes from *Cupriavidus necator* (blue) were expressed on the pTrc99A plasmid. In some experiments either the *tesB* or the *yciA* gene from *E. coli* coding thioesterases (blue) were expressed on the pACYC184 plasmid. These thioesterases were intended to hydrolyze 3-hydroxybutyryl-CoA into 3-hydroxybutyrate, but might also hydrolyze other thioesters (including acetyl-CoA into acetate)

As a model organism, *Escherichia coli* has been proposed for the generation of 3-HB by overexpressing heterologous pathways. One pathway to 3-HB involves the enzymes acetyl-CoA C-acetyltransferase or β-ketothiolase ([EC 2.3.1.9], coded by the *phaA* gene) and acetoacetyl-CoA reductase ([EC 1.1.1.36], *phaB*) (Fig. 1). Expressing these genes from *Cupriavidus necator* in *E. coli* resulted in about 0.4 g/L 3-HB in shake flask culture [1]. Inclusion of *ptb* (phospho-transbutyrylase) and *buk* (butyrate kinase) from *Clostridium acetobutylicum*, allowing formation of ATP through the intermediate of 3-hydroxybutyryl-phosphate, led to 5 g/L 3-HB in a glucose-supplemented complex medium in shake flask culture and 12 g/L 3-HB with 8.5 g/L acetate in a fed-batch process [1]. Addition of acetate in *E. coli* expressing the genes *phaA*, *phaB*, and *pct* (encoding propionyl-CoA transferase) attained titers of 5.2 g/L and a productivity of 0.22 g/L·h in test tube cultures [4]. In another study, overexpression of *phaA*, *phaB*, and *tesB* (coding a thioesterase) led to 3.6 g/L 3-HB in 48 h with a 0.41 g/g yield in glucose-supplemented complex medium [5], while fed-batch culture of this strain accumulated 12.2 g/L 3-HB at 0.5 g/L·h [6]. In comparison, under microaerobic conditions, a native producer *Halomonas* sp. generated 15.2 g/L 3-HB [7]. A nitrate fed-batch process using *Halomonas* sp. in which the stored poly(3-hydroxybutryate) was degraded to 3-HB led to 40.3 g/L at 0.48 g/L·h in shake flask studies under microaerobic conditions [2].

Acetyl-CoA is the key precursor for 3-HB, and several native pathways which metabolize acetyl-CoA compete with the introduced 3-HB pathway. Most strategies for 3-HB production also generate acetate as a by-product [1, 8]. Deletion or repression of *pta-ackA* or *poxB* genes are common approaches to reduce acetate formation, and this strategy has previously also been applied to several acetyl-CoA-derived products such as citramalate [9], butyric acid [10], *n*-butanol [11], as well as 3-HB [12]. However, the primary metabolic competition for acetyl-CoA is the enzyme citrate synthase (*gltA* gene), through which 63% of the acetyl-CoA flows during steady-state, glucose-limited culture [13]. Although outright deletion of citrate synthase increases the yield of acetyl-CoA-derived products from glucose, including citramalate [9] and mevalonate [14], this deletion prevents *E. coli* growth on glucose as the sole carbon source, necessitating a medium supplement such as glutamate [15]. Alternatively, approaches have been proposed to reduce rather than eliminate citrate synthase flux. For example, decreasing the *gltA* promoter strength increased intracellular oxaloacetate and acetyl-CoA concentrations, leading to improved lysine production in *Corynebacterium glutamicum* [16]. Reducing the activity of citrate synthase directly using targeted amino acid substitutions lowered k_CAT_ and increased K_M_, resulting in the diversion of carbon from the TCA cycle to the model compound acetate [17], as well as to citramalate [18].

Nutrient limitation can also have a profound effect on the formation of organic products such as 3-HB. During an N-limited fed-batch cultivation on mixed sugars, *E. coli* overexpressing the *t3* (β-ketothiolase) and *rx* (acetoacetyl-CoA reductase) genes from *Halomonas boliviensis* generated 1.8 g/L 3-HB and 2.7 g/L acetate [8]. An N-limited exponential-linear feed process with *E. coli* expressing *t3* and *rx* genes led to 4.1 g/L 3-HB and 7 g/L acetate, while a P-limited exponential-linear process yielded 6.8 g/L 3-HB and 9 g/L acetate [19]. A combination of repeated batch followed by N-starved process with *E. coli* additionally overexpressing the *zwf* gene intended to increase flux through the NADPH-generating pentose phosphate pathway led to 12.7 g/L 3-HB in *E. coli* AF1000 [20] and 16.3 g/L in *E. coli* BL21 [12]. More recently, overexpression of another thioesterase (*yciA* gene) along with the *zwf, t3*, and *rx* genes in a repeated batch followed by N-starved process generated 14.3 g/L 3-HB in *E. coli* AF1000 strain [21].

The goal of this study was to use citrate synthase variants for the formation of acetyl-CoA-derived 3-HB. We propose that creating a metabolic restriction at the intermediate acetyl-CoA could divert more acetyl-CoA towards 3-HB. We also examined the effect of overexpressing each of two commonly used thioesterases, TesB or YciA, as well as pantothenate kinase (CoaA).

## Results

### Growth rates of GltA variant strains

This study compared a strain expressing a wild-type citrate synthase (MEC1365), a citrate synthase knockout (MEC1381), and five citrate synthase variants (Table 1) using *E. coli* W, a strain of *E. coli* which is able to metabolize sucrose [22, 23]. The citrate synthase variants each contained a single amino acid substitution which has previously been shown to decrease citrate synthase activity in *E. coli* [17, 18, 24]. The variants are MEC1394 (containing the A267T substitution), MEC1410 (F383M), MEC1411 (K167A), MEC1482 (V361A) and MEC1567 (M372S). All strains also contained the *ldhA*, *poxB*, and *pta-ackA* deletions to mitigate carbon loss due to acetate production [25]. To highlight that the only difference between the strains is the amino acid sequence of the chromosomally expressed citrate synthase protein, the strains will be referred to by their amino acid substitution (e.g., GltA[K167A]) rather than by their strain name (e.g., MEC1411). MEC1365 expressing the wild-type citrate synthase attained a growth rate of 0.580 ± 0.008 h^− 1^ with the production of pyruvate (0.016 ± 0.002 g/g) and acetate (0.004 ± 0.007 g/g). Compared to the wild-type GltA strain, the GltA variant strains showed 15–25% slower growth rates (0.43–0.50 h^− 1^) and, except for GltA[M372S], greater pyruvate and acetate yields (Figure S1). Though acetate yields were not significantly different among the five GltA variants, the GltA[A267T] variant accumulated nearly double the pyruvate as the other variants. The growth rate of MEC1381 (Δ*gltA*) was not examined because this strain is unable to grow on glucose as the sole carbon source.

**Table 1:** Strains used in this study

Strain	Relevant characteristics	Reference
ATCC 9637	*Escherichia coli* W	Wild-type
MEC1316	ATCC 9637 Δ*poxB* Δ*ldhA*	[79]
MEC1353	ATCC 9637 Δ*gltA*	This study
MEC1365	MEC1316 Δ*poxB* Δ*ldhA* Δ*pta-ackA*	This study
MEC1381	MEC1365 Δ*gltA*	This study
MEC1394	MEC1381 Δ*gltA*::*gltA*^[A267T]^	This study
MEC1410	MEC1381 Δ*gltA*::*gltA*^[F383M]^	This study
MEC1411	MEC1381 Δ*gltA*::*gltA*^[K167A]^	This study
MEC1482	MEC1381 Δ*gltA*::*gltA*^[V361A]^	This study
MEC1567	MEC1381 Δ*gltA*::*gltA*^[M372S]^	This study

### Production of 3-HB by GltA variant strains

**Fig. 2:**
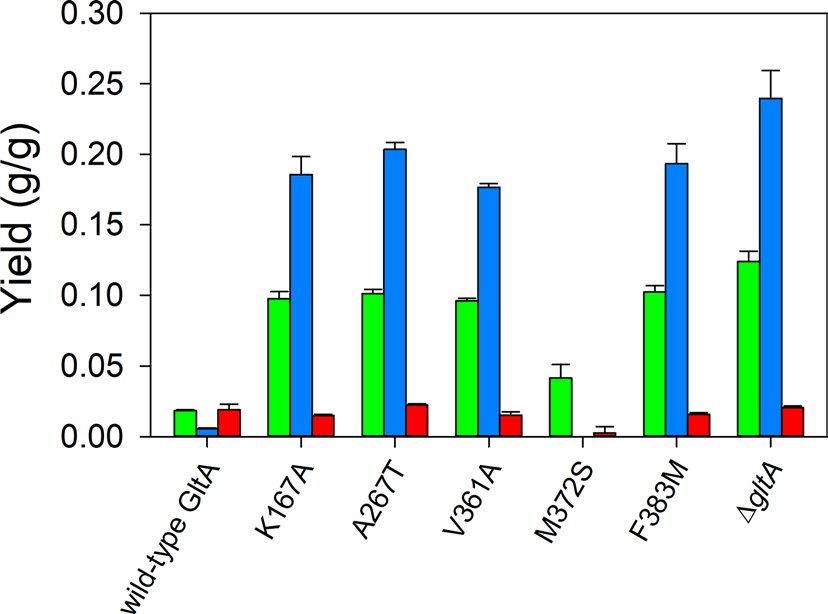
Effect of citrate synthase (GltA) substitutions on product yields using 8 g/L glucose as the sole carbon source in shake flask culture. Yields of 3-hydroxybutyrate (green), pyruvate (blue) and acetate (red) are shown for variants containing the plasmid pHR-AB. All strains have deletions in *poxB*, *ldhA*, *pta* and *ackA* genes. The Δ*gltA* strain was supplemented with 1 g/L glutamate

Plasmid pHR-AB expressing *phaA* encoding β-ketothiolase and *phaB* encoding acetoacetyl-CoA reductase (Fig. 1) was transformed into all *E. coli* strains to evaluate 3-HB production in shake flask culture. In a first study, the conversion of 3-hydroxybutyryl-CoA to 3-hydroxybutyrate was assumed to be mediated by native thioesterases. Shake flasks were conducted at 30 °C using glucose as the sole carbon source, except cultures of MEC1381 (Δ*gltA*) which was supplemented with glutamate (Fig. 2, Table S1). Shake flasks and similar low volume processes invariably suffer from limited oxygenation and minimal pH control. In this cases, strains, which included one or more plasmids, would be expected to grow at differing rates (Figure S1). Therefore, samples were taken after 6 h of growth to minimize the potential for consumption of 3-HB, and product yield from glucose consumed was used as the criterion for comparing strains. MEC1365 (wild-type GltA) generated the lowest 3-HB (0.02 ± 0.00 g/g) and pyruvate (0.01 g/g ± 0.00 g/g) yields. Four GltA variant strains generated significantly greater 3-HB (0.10 ± 0.00 g/g) than the wild-type, although no significant difference was observed between the four variants (K167A, A267T, V361A, F383M). These four GltA variant strains also accumulated more pyruvate (> 0.18 g/g) than MEC1365. MEC1567 (GltA[M372S]) produced 0.04 ± 0.01 g/g 3-HB, significantly greater than MEC1365 but less than the other four variants. The GltA[M372S] variant did not accumulate pyruvate (Fig. 2, Table S1). The Δ*gltA* knockout strain produced the greatest 3-HB (0.12 ± 0.01 g/g) and pyruvate (0.24 ± 0.02 g/g).

### Production of 3-HB by GltA variant strains expressing thioesterases

Accumulation of pyruvate in GltA variants expressing *phaA*/*phaB* suggested a bottleneck between pyruvate and acetyl-CoA, a step mediated in *E. coli* by the pyruvate dehydrogenase complex under aerobic conditions. One potential cause of pyruvate accumulation could be a limited conversion of 3-hydroxybutyryl-CoA to 3-HB by native thioesterases. In addition to potentially reducing flux through the reversible steps of this introduced pathway, a bottleneck in this final conversion could limit the availability of CoASH which acts as a substrate for pyruvate dehydrogenase. To test whether pyruvate accumulation could be attributed to insufficient conversion to 3-HB, native acyl-CoA hydrolases (EC 3.1.2.20) coded by the *tesB* gene [5] or the *yciA* gene [21] were each individually overexpressed in a low copy number plasmid under a constitutive promoter J23107 and introduced into strains containing the pHR-AB plasmid. One potential consequence of overexpressing any thioesterase, however, is the direct conversion of acetyl-CoA into acetate (Fig. 1).

**Fig. 3:**
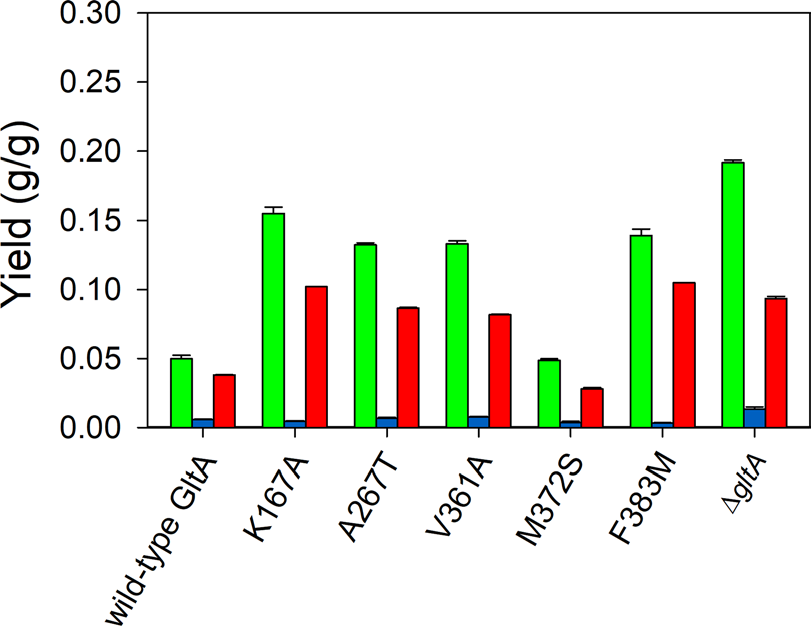
Effect of TesB expression on product yields using 8 g/L glucose as the sole carbon source in shake flask culture using GltA variant strains. Yields of 3-hydroxybutyrate (green), pyruvate (blue) and acetate (red) are shown for variants containing the plasmids pHR-AB and pHR-tesB. All strains have deletions in *poxB*, *ldhA*, *pta*, and *ackA* genes. The Δ*gltA* strain was supplemented with 1 g/L glutamate

TesB is a native promiscuous thioesterase in *E. coli* that mediates the irreversible one-step hydrolysis of short-chain acyl-CoAs [26]. In shake flask experiments, overexpressed TesB in MEC1365 expressing the wild-type citrate synthase resulted in a 2.5× increase in 3-HB yield (0.05 ± 0.00 g/g) and a 2.0× increase in acetate yield (0.04 ± 0.00 g/g). In GltA variant strains and the Δ*gltA* knockout strain, overexpression of TesB significantly decreased pyruvate accumulation but consistently increased acetate formation about 5-fold (Fig. 3, Table S1). The Δ*gltA* knockout strain MEC1381 showed the greatest 3-HB yield (0.19 ± 0.00 g/g), 60% greater than the yield observed in absence of TesB overexpression. The GltA[K167A] variant attained the greatest 3-HB yield (0.16 ± 0.00 g/g) among the variants, about 60% greater than observed in the same strain without overexpression of TesB (*p* < 0.05). The GltA[M372S] variant showed an identical 3-HB yield (0.05 ± 0.00 g/g) as the wild-type citrate synthase strain MEC1365 (Fig. 3).

**Fig. 4:**
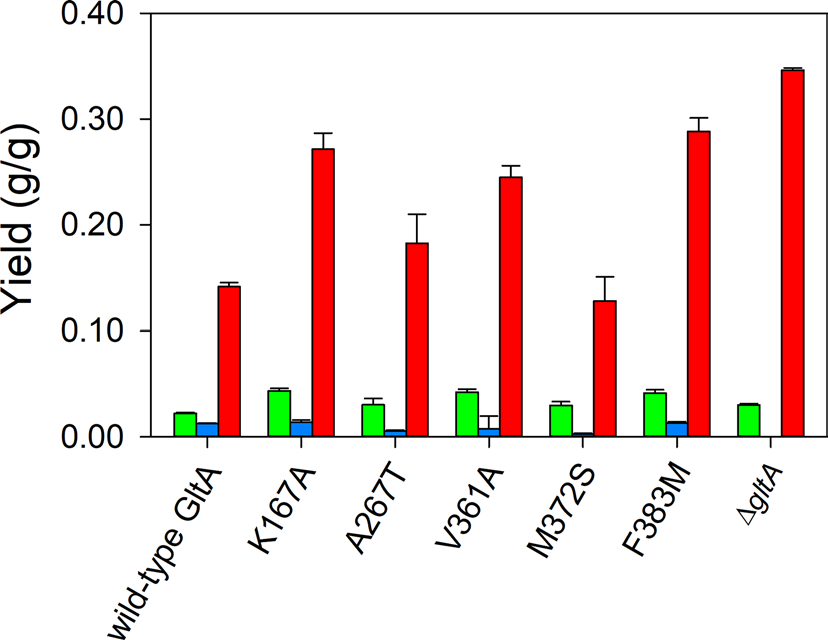
Effect of YciA expression on product yields using 8 g/L glucose as the sole carbon source in shake flask culture using GltA variant strains. Yields of 3-hydroxybutyrate (green), pyruvate (blue) and acetate (red) are shown for variants containing the plasmids pHR-AB and pHR-yciA. All strains have deletions in *poxB*, *ldhA*, *pta*, and *ackA* genes. The Δ*gltA* strain was supplemented with 1 g/L glutamate

YciA is one of the seven “hot dog” fold thioesterases in *E. coli*, containing a conserved fold of a five-stranded anti-paralled β-sheet wrapped around an elongated α-helix [27]. Similar to TesB, YciA is a promiscuous thioesterase with substrates ranging from short to long-chain acyl-CoAs since hot dog fold structures lack defined non-solvated binding pockets and a conserved catalytic residue leading to multiple catalytic residues [28]. When YciA was overexpressed in the Δ*gltA* and GltA variants which also contained the pHR-AB plasmid, the formation of both pyruvate and 3-HB was significantly diminished (Fig. 4, Table S1). For example, the GltA[A267T] variant expressing pHR-AB generated 0.10 g/g 3-HB and 0.20 g/g pyruvate in the absence of YciA (Fig. 2), but only 0.03 g/g 3-HB and 0.006 g/g pyruvate with YciA overexpression. Instead, overexpression of YciA resulted in 7–18× greater acetate formation (*p* < 0.05): for example, the GltA[A267T] variant expressing pHR-AB generated 0.02 g acetate/g glucose (Fig. 2), while this variant additionally expressing pHR-yciA generated 0.18 g acetate/g glucose (Fig. 4). The greatest acetate yield was attained by the Δ*gltA* knockout strain MEC1381 (0.35 ± 0.00 g/g).

The overexpression of either thioesterase, TesB or YciA, did significantly diminish pyruvate accumulation in 3-HB generating *E. coli* GltA variants. However, in both cases thioesterase overexpression simultaneously increased the formation of acetate. While overexpression of these thioesterases was anticipated to improve the conversion of 3-hydroxybutyryl-CoA into 3-HB, both thioesterases likely converted acetyl-CoA directly into acetate and thereby limited the flux into the 3-HB pathway (Fig. 1). In the case of TesB, decreased pyruvate was balanced by increased 3-HB and acetate, whereas in the case of YciA, acetate formation increased at the expense of pyruvate and 3-HB. These results suggest that buildup of acetyl-CoA was indeed a factor in pyruvate accumulation in GltA variants. Additional research is necessary to determine whether modulating the expression of these thioesterases or expressing a thioesterase with specific activity towards 3-hydroxybutyryl-CoA would benefit 3-HB formation.

### Production of 3-HB by GltA variants strains expressing pantothanate kinase

**Fig. 5:**
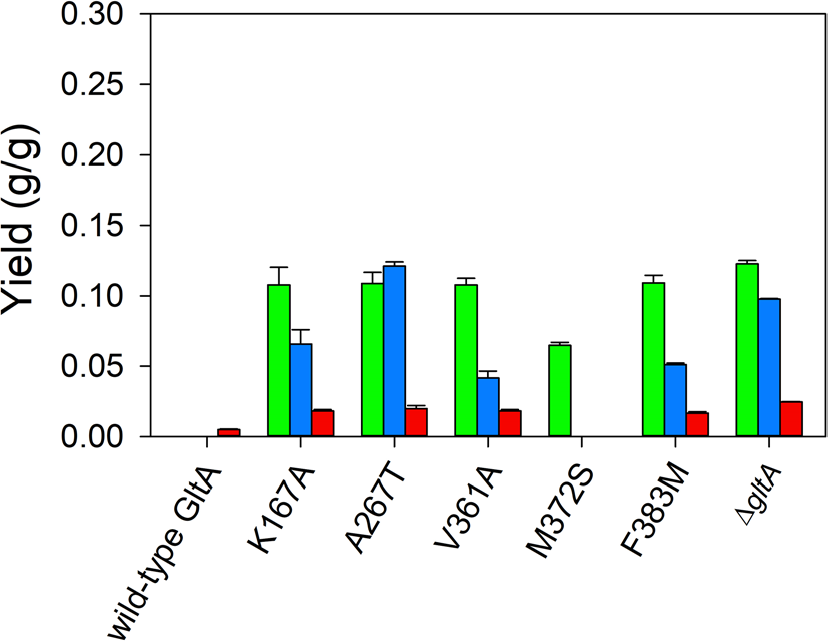
Effect of CoaA expression on product yields using 8 g/L glucose as the sole carbon source with 1 mM pantothenate in shake flask culture using GltA variant strains. Yields of 3-hydroxybutyrate (green), pyruvate (blue) and acetate (red) are shown for variants containing the plasmid pHR-AB. All strains have deletions in *poxB*, *ldhA*, *pta* and *ackA* genes. The Δ*gltA* strain was supplemented with 1 g/L glutamate

Considering the central role of the conversion of pyruvate to acetyl-CoA in the formation of 3-HB in GltA variants, we next examined the effect of overexpressing pantothenate kinase with medium supplementation of pantothenate (vitamin B_5_) on 3-HB formation. Pantothenate kinase (CoaA or PanK) mediates the ATP-dependent conversion of pantothenate to 4P-pantothenate as the first step in the CoASH biosynthetic pathway. The enzyme is inhibited by intracellular CoASH and its thioesters [29]. Overexpression of CoaA did result in significantly lower pyruvate accumulation in all strains examined (Fig. 5, Table S1). For example, the GltA[A267T] variant with overexpressed CoA generated 0.121 ± 0.003 g/g pyruvate, 40% less than the same variant with only native CoaA expression (*p* < 0.05). This decreased formation of pyruvate was not however accompanied by a concomitant increase in 3-HB or acetate formation.

### Batch processes

**Fig. 6:**
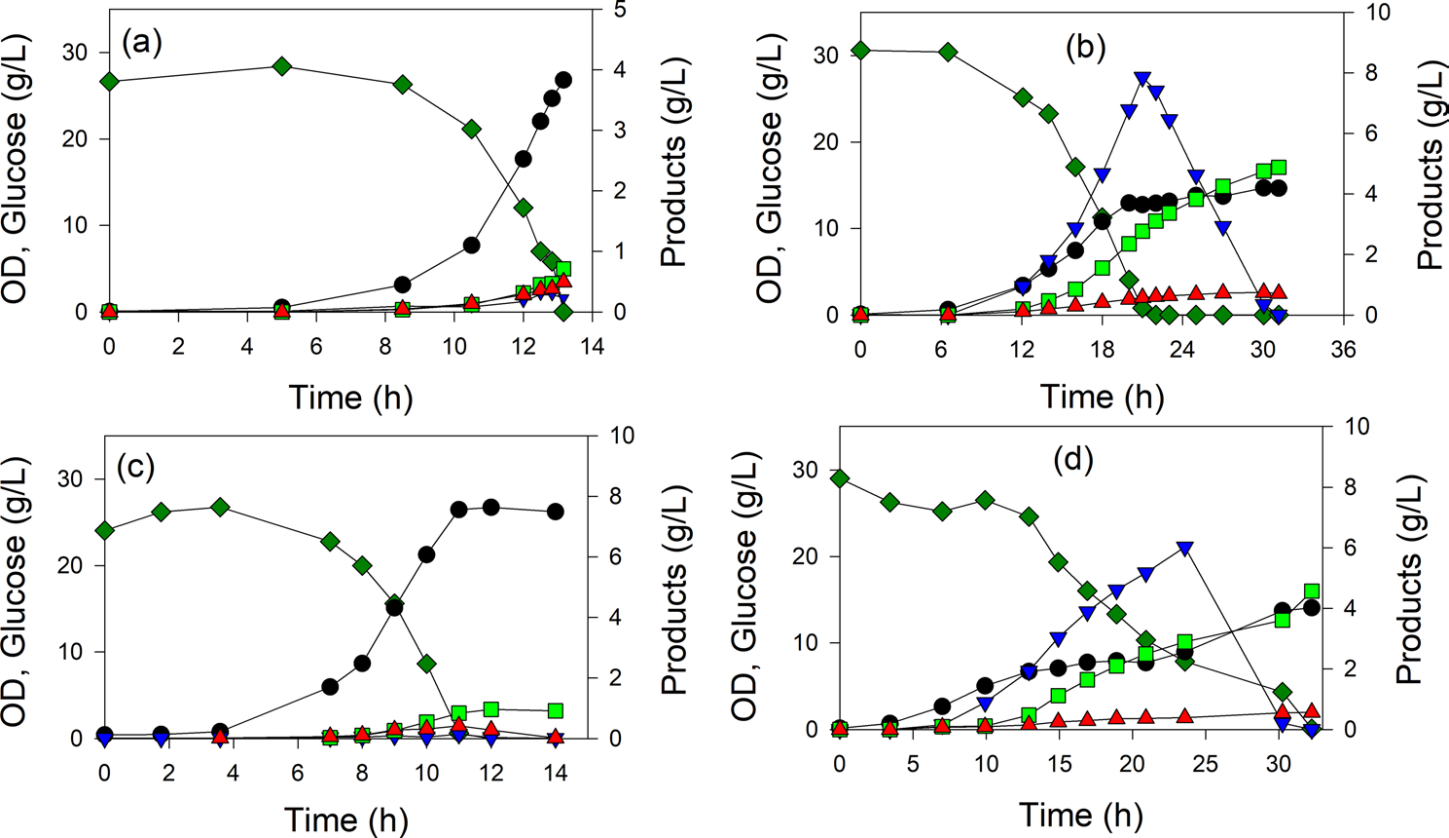
Controlled growth of W Δ*ldhA* Δ*poxB* Δ*pta-ackA* strains expressing different chromosomal *gltA* genes coding citrate synthase enzymes in defined medium with 30 g/L glucose and 25 mM MOPS, induced with 50 µM IPTG. Each strain expressed genes in the 3-HB pathway via plasmid pHR-AB in 1.25 L fermenter. Concentrations of glucose (dark green), pyruvate (blue), OD (•), acetate (red), 3-HB (bright green) were measured during the course of the 1.25 L batch processes. (a) wild-type GltA, (b) GltA[A267T], (c) GltA[M372S], (d) GltA[K167A]

Shake flask studies were conducted to screen GltA variants for 3-HB formation, though such cultures have several disadvantages including absence of pH control necessitating a high buffer concentration, inability to use high glucose concentration, and lack of oxygen mass transfer to support growth to a high cell density. Also, in the shake flask studies samples were taken early during growth before glucose was depleted to ensure 3-HB was not re-assimilated. We therefore next conducted prolonged batch studies with 30 g/L glucose as the sole carbon source, first selecting MEC1365 (wild-type GltA), and the variants GltA[A267T], GltA[M372S] and GltA[K167A]. Each strain was transformed the plasmid pHR-AB expressing the *phaA* and *phaB* genes coding for enzymes in the 3-HB pathway (Fig. 1). MEC1365 generated 0.71 g/L 3-HB with the productivity of 0.054 g/L·h and also accumulated 0.23 g/L pyruvate (Fig. 6a). The GltA[A267T] variant produced 4.9 g/L 3-HB with a productivity of 0.14 g/L·h and yield of 0.17 g/g (Fig. 6b). This variant also accumulated about 8.8 g/L pyruvate, which beginning at the time of glucose depletion (22 h), was itself metabolized for additional biomass and 3-HB formation. GltA[M372S] generated 1.0 g/L 3-HB at the rate of 0.08 g/L·h and yield of 0.041 g/g (Fig. 6c). The GltA[K167A] variant MEC1411 generated 4.6 g/L 3-HB with a yield of 0.16 g/g (Fig. 6d). Similar to GltA[A267T], 6.0 g/L pyruvate accumulated and was quickly metabolized at the time of glucose depletion. In all four strains acetate accumulated to less than 0.4 g/L.

Overexpression of TesB in general improved 3-HB yield in shake flask culture (Fig. 3 versus Fig. 2), though pyruvate formation was greatly diminished. Of the five variants studied using TesB, the GltA[K167A] variant achieved the greatest yield in shake flask at 0.15 g/g (Fig. 3). We therefore conducted additional batch processes using the wild-type GltA strain MEC1365 and the GltA[K167A] variant MEC1411, both expressing *phaA*, *phaB*, and *tesB* on two plasmids. Although no pyruvate was generated in either case, MEC1365 generated 1.5 g/L 3-HB and 1.7 g/L acetate (Figure S2a), while GltA[K167A] generated 4.3 g/L 3-HB and 3.2 g/L acetate (Figure S2b). Surprisingly, in prolonged batch processes, TesB overexpression did not improve 3-HB yield or titer compared to strains not overexpressing TesB. For the GltA[K167A] variant not overexpressing TesB, much of the accumulated pyruvate was converted to 3-HB after glucose was depleted (Fig. 6b). For the GltA[K167A] variant overexpressing TesB, acetate instead accumulated, and this biochemical was not converted into 3-HB after glucose depletion (Figure S2b). These results demonstrate that the shake flask experiments do not directly predict results in a controlled bioreactor having a greater glucose concentration and operating for a longer duration. For a subsequent study using a repeated batch, we selected strains which relied only on native thioesterases (i.e., did not overexpress TesB) to minimize acetate accumulation.

### Repeated batch processes

**Fig. 7:**
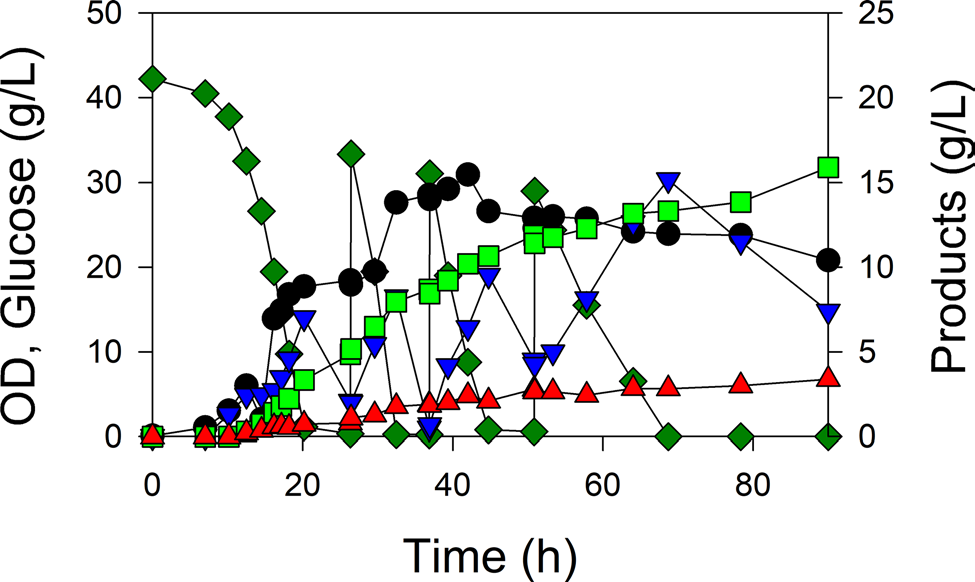
Controlled repeated batch growth MEC1394 (W Δ*ldhA* Δ*poxB* Δ*pta-ackA* Δ*gltA*::gltA^[A267T]^) containing plasmid pHR-AB in a defined medium supplemented with 40 g/L glucose and 25 mM MOPS, induced with 50 µM IPTG. A 52 mL solution of 39 g glucose and 50 mg ampicillin was added three times during the repeated batch. The medium was designed to become depleted in phosphorus when the OD reached 30. Concentrations of glucose (dark green), pyruvate (blue), OD (•), acetate (red), 3-HB (bright green) were measured during the course of the processes

MEC1394 (GltA[A267T])/pHR-AB generated the greatest 3-HB titer and yield in the batch process (Fig. 6b), and therefore was selected for a high cell density fermentation which became phosphorus starved. Because pyruvate accumulated and itself was consumed after glucose was depleted (Fig. 6b), a repeated batch process was performed, whereby glucose was added three times approximately after both glucose and pyruvate were consumed. GltA[A267T] produced 15.9 g/L 3-HB (effective concentration of 21.3 g/L without dilution from pH control and feeding) with an overall yield of 0.16 g/g glucose and 0.23 g/L·h overall productivity (Fig. 7). The glucose consumption rate decreased with each glucose addition: 5.9 g/L·h, 3.9 g/L·h, and 1.6 g/L·h during the final period. Acetate was continuously generated and reached a 3.4 g/L final concentration.

## Discussion

The goal of this study was to assess targeted substitutions in citrate synthase (GltA) and their affect on the generation of an acetyl-CoA derived product, (R)-3-hydroxybutyric acid (3-HB). Citrate synthase is a key enzyme in central metabolism at the entry of acetyl-CoA into the TCA cycle which serves as a metabolic control site between central carbon metabolism and biomass synthesis [30, 31]. Type II citrate synthases form hexamers which are allosterically regulated by NADH [32–34]. The structure and function of the *E. coli* enzyme has been extensively studied, typically using site-directed mutagenesis to understand important residues associated with the active site as well as allosteric inhibition by NADH [24, 35–38]. For example, the F383 residue is highly conserved in Type II enzymes and forms an “unusual edge-on interaction” with the acetyl group of acetyl-CoA [37, 39]. The F383A substitution leaves a large cavity which destabilizes the active site, reducing k_CAT_ by 97% [37], while the less severe F383M substitution decreases k_CAT_ by 93% [18]. Similarly, the K167A substitution weakens allosteric binding for NADH and discourages hexamer formation [24]. The K167A variant increases K_M_ for acetyl-CoA by 2× and K_M_ for NADH by 2.6× but does not change the K_M_ for oxaloacetate and k_CAT_ [24]. In absence of acetyl-CoA, residues 267–297 possess high thermal factors indicating mobility, while in the presence of acetyl-CoA this mobile loop undergoes considerable conformational changes [40]. Substitutions in this mobile loop can affect both catalytic activity and acetyl-CoA affinity [17, 40, 41]. For example, the A267T substitution increases apparent K_M_ for acetyl-CoA by 2.3× and decreases k_CAT_ from 34 s^− 1^ to 2.1 s^− 1^ [17]. In this work, we selected several citrate synthase substitutions characterized in previous studies [17, 18, 24] to modulate the activity of the chromosomally expressed enzyme. Altering citrate synthase is hypothesized to affect *E. coli* physiology and alter the availability of acetyl-CoA for the production of 3-HB: A267T (located in the mobile loop proximal to the active residue H264), F383M (involved in stabilization of acetyl-CoA binding), K167A (affecting NADH and acetyl-CoA binding), V361A, and M372S (located in the α-helix adjacent to catalytic residue D362). Residues F383, K167, A267, and V361 are highly conserved in Type II citrate synthase [41, 42]. Therefore, these substitutions likely would have similar effects on other prokaryotes expressing a Type II enzyme and which may be of interest for the production of any acetyl-CoA-derived biochemical.

### Altering citrate synthase affects growth rate and by-product formation

Citrate synthase variants having substitutions close to the active site (F383M, A267T, V361A) or involved in NADH (K167A) or acetyl-CoA binding (F383M, A267T, K167A) affect cell physiology, and this study with a different strain was consistent with previous work [17, 18]. The maximum specific growth rates of all variants were 13–25% lower than MEC1365 expressing the wild-type citrate synthase (Figure S1). The GltA[M372S] variant showed the highest growth rate among the variants (only 13% lower than MEC1365). The GltA[M372S] variant showed results very similar to the wild-type GltA strain MEC1365 (e.g., Fig. 2), a result which is not surprising since this variant was previously shown to have a minor impact on wild-type *E. coli* growth and acetate formation [17]. Though V361 and M372 are located on the same α-helix and neither is involved in catalytic activity or substrate binding, the GltA[V361A] variant showed a 21% lower specific growth rate than MEC1365, probably because of the proximity of V361 to the catalytic residue D362 [41]. Nevertheless, our results provide further evidence that any restriction of flux through citrate synthase with the aim of improving acetyl-CoA availability for the production of biochemicals will also lead to a strain having a lower growth rate on glucose as the sole carbon source.

Because of the *poxB pta-ackA* deletions in all examined strains, cells would be expected not to accumulate acetate, and indeed less than 0.005 g/g acetate was observed in MEC1365 (Figure S1). However, all variants except GltA[M372S] surprisingly showed modest, but significant, acetate (Figure S1). For example, the GltA[A267T] variant produced 0.15 g/g pyruvate and 0.022 g/g acetate. Although the route for acetate formation is uncertain given the absence of phosphate acetyltransferase and pyruvate oxidase, clearly the introduction of modifications in citrate synthase creates a metabolic bottleneck at acetyl-CoA which leads to accumulation of both pyruvate and acetate.

### Altering citrate synthase affects 3-HB production

Citrate synthase has long been recognized as a key conduit controlling entry into the TCA cycle, and genetic tools have sought to modify citrate synthase expression/activity to increase or decrease the carbon flow to the TCA cycle. For example, placing expression under an inducible promoter [43], engineering a less active promoter [16], using mRNA based lysine riboswitches [44], artificial micro RNA inhibition [45] and CRISPRi-based inhibition [46] have been used in several organisms in the context of improving formation of acetyl-CoA-derived products such as butanol and poly(hydroxybutyrate) or oxaloacetate-derived products such as lysine. Some of these strategies may be limited by lack of scalability and need for medium supplementation. An outright deletion of GltA does improve formation of acetyl-CoA-derived products (e.g., Fig. 2), but also requires supplementation with glutamate or another TCA cycle intermediate [9, 14, 15]. The chromosomal GltA variants used in the current study are stable and can be grown without medium supplementation.

In shake flask culture, four of the five citrate synthase variants showed > 5-fold increase in 3-HB yield from glucose compared to MEC1365 expressing the wild-type enzyme, while GltA[M372S], which also attained the highest growth rate of the variants, showed a 2.2-fold increase in 3-HB generation (Fig. 2). The very low 3-HB yield in the strain expressing the wild-type citrate synthase is suprising, but may be due to the selection of the sucrose-consuming W strain, previously shown to be suboptimal for 3-HB formation [12]. In controlled batch culture, the GltA[A267T] variant formed 3-HB at a yield of 0.17 ± 0.01 g/g from glucose as the sole carbon source (Fig. 6b), equivalent to the yield reported previously when both *zwf* and *yciA* were overexpressed [21]. The use of GltA variants offers a complimentary strategy to increase yield of acetyl-CoA-derived products during unrestricted growth without overexpressing genes other than those involved in the synthetic pathway (i.e., in this case *phaA* and *phaB*).

The 3-HB production appears to be inversely proportional to growth rate (Figure S1; Fig. 2) and correlates with the severity of citrate synthase substitutions studied previously for growth and acetate production [17]. The GltA[K167A] variant in *Klebsiella pneumoniae* increased 1,3-propanediol yield from glycerol by only 10% though biomass and acetate yields, and growth rate, were not significantly affected [47]. Interestingly, the stated goal in that work was to *increase* the activity of citrate synthase using the K167A substitution by *reducing* the NADH binding during a microaerobic process. In contrast in our current work, GltA[K167A] decreased growth rate by 20% but increased 3-HB yield by a factor of 5 under fully aerobic conditions. These results with *E. coli* suggest that diminished NADH-binding in GltA[K167A] which might increase citrate synthase flux under aerobic conditions is much less than the effect of reduced acetyl-CoA binding [24]. Though different microbes are being compared, the evidence remarkably suggests GltA[K167A] could enhance citrate synthase flux under anaerobic conditions but reduce citrate synthase flux under aerobic conditions, and thus examining product formation using the K167A substitution under various levels of oxygenation would be of great interest. Note that the 1,3-propanediol pathway is biochemically separated from citrate synthase, while the 3-HB pathway commences with two moles of acetyl-CoA. Finally, since the K167A substitution affects hexamer formation [24], this substitution may also play a role in the ability of citrate synthase to participate in multienzyme complexes which channel substrates, and which might also differ between aerobic and anaerobic conditions [48].

### Pyruvate accumulation suggests a metabolic bottleneck

We encountered significant pyruvate accumulation in most GltA variants during the cell growth rate studies (i.e., without the 3-HB pathway), as well as in the context of 3-HB production. Numerous studies focused on products from acetyl-CoA have also reported pyruvate accumulation. For example, deletion of *pta-ackA* genes with the goal of ethanol production led to a 30-fold increase in pyruvate accumulation compared to the strain without these knockouts [49], while pyruvate also accumulated as a consequence of the *pta* knockout during tryptophan [50] and valine [51] production and *pta* and *poxB* knockouts in 3-HB production [12]. Although maintaining the presence of the *pta-ackA* genes largely prevents pyruvate accumulation during the formation of an acetyl-CoA-derived product, acetate then accumulates with no benefit to the desired product [12, 52]. Accumulation of pyruvate by the deletion of the *pta* gene has been attributed to intracellular CoASH imbalance [53]. In the current study, though modest pyruvate was observed in MEC1365 with wild-type GltA, pyruvate formation reached 0.20 g/g in variants and 0.23 g/g for the Δ*gltA* strain (Fig. 2), suggesting a bottleneck for the variants which could be due to a variety of factors related to citrate synthase. One hypothesis explored was that, because CoASH regeneration via citrate synthase is diminished in variants, the hydrolysis of 3-hydroxybutyryl-CoA to CoASH limited CoASH availability for the conversion of pyruvate to acetyl-CoA via the pyruvate dehydrogenase complex.

To improve the hydrolysis of 3-hydroxybutyryl-CoA we compared overexpression of two native *E. coli* thioesterases, TesB and YciA. These thioesterases have been used in a wide range of studies, including the formation of adipic acid [54], butyrate [10] and 3-hydroxyvalerate [55]. Generally understood to have broad specificity to C_5_ – C_14_ acyl-CoAs [56] and 3-hydroxyacyl-CoA esters [57], the presence of TesB previously improved 3-HB formation without acetate accumulation [6]. In the current study in shake flasks, overexpression of TesB did increase 3-HB yield slightly and essentially eliminated pyruvate formation, however, acetate accumulation increased dramatically (Figs. 2 and 3). For example, overexpression of TesB in the GltA[K167A] variant with the 3-HB pathway led to a 58% increase in 3-HB formation but a 5.8-fold increase in acetate compared to GltA[K167A] expressing TesB at the native activity. Inexplicitly, in the controlled bioreactor the improvement in 3-HB yield observed in the shake flasks disappeared while acetate accumulation was even greater (Figure S2b). Specifically, under unrestricted growth in the bioreactor using a medium with a greater concentration of glucose, overexpression of TesB in GltA[K167A] led to slight decrease in 3-HB (4.3 g/L versus 4.6 g/L without TesB), but led to much greater acetate formation (3.2 g/L versus 0.3 g/L without TesB). Thus, overexpression of TesB did eliminate a bottleneck of pyruvate formation in variants caused by curtailing citrate synthase activity, but TesB merely shifted the product to acetate. Clearly, for improved 3-HB formation, a careful tuning between the hydrolysis of acetyl-CoA and 3-hydroxybutyryl-CoA is necessary.

The thioesterase YciA was also examined in shake flask cultures with all the variants. YciA possess lower affinity (2.45× K_M_) and catalytic activity (90% lower) towards acetyl-CoA compared to higher chain carbon acyl groups like butyryl-CoA [27]. Moreover, previous research showed no increased acetate formation during 3-HB with overexpressed YciA [21]. Surprisingly, in the current study overexpression of YciA led a 20-fold increase in acetate formation at the expense of 3-HB (Fig. 4). Thus, we found YciA behaved much like TesB in eliminating the bottleneck of pyruvate accumulation, but performed worse as a consequence of shifting the acetyl-CoA derived product from 3-HB to acetate. We interpret these results to be a consequence of a significant increase in acetyl-CoA pool in citrate synthase variants. A much greater intracellular acetyl-CoA concentration could result in this thioester being the predominate reaction substrate, regardless of the relative kinetics of these thioesterases (i.e., acetyl-CoA compared to 3-hydroxybutyryl-CoA). Thus, the results with both TesB and YciA suggest value in using a thioesterase with either lower expression or low affinity for acetyl-CoA. Additional research, preferably at steady-state conditions, can clarify how the citrate synthase variants impact intracellular concentrations.

### Pantothenate kinase overexpression decreases pyruvate accumulation but does not improve 3-HB production

Another approach studied to reduce pyruvate accumulation was to increase the total CoASH pool. The approach of reducing citrate synthase activity would be expected to promote accumulation of acetyl-CoA [18], which is a potent inhibitor of pyruvate dehydrogenase [58], leading to pyruvate accumulation. One way to improve CoASH availability directly is by the overexpression of pantothenate kinase (CoaA), which is the rate limiting step in CoASH biosynthesis [59]. Previous research has confirmed that the total CoASH/acetyl-CoA concentration is increased by overexpression of this enzyme [60]. Moreover, overexpression of pantothenate kinase has been studied before to improve acetyl-CoA derived products including isoamyl acetate [61], succinate [62], and fatty acids [63]. When we overexpressed CoaA on a low copy number plasmid with pantothenate supplementation, pyruvate accumulation indeed did diminish in all the GltA variants by about 40–75% compared to the same variants with only native expression of CoaA, although both 3-HB and acetate formation were not significantly changed (Figs. 2 and 5). The pantothenate kinase from *E. coli* is tightly controlled by CoAs and acyl-CoAs [28, 64], and therefore the enzyme could be suppressed in variants in which the acetyl-CoA pool is expected to be elevated. Nevertheless, the reduction in pyruvate accumulation demonstrates that overexpressed CoaA did relieve the bottleneck between pyruvate and acetyl-CoA, and demonstrates that the accumulation of pyruvate is a consequence of insufficient CoASH and/or accumulation of acetyl-CoA. The observation that 3-HB formation did not benefit from CoaA overexpression, despite the reduction of pyruvate, suggests that the activity of β-ketolase remains insufficient to direct the potential increased availability of acetyl-CoA into the 3-HB pathway. Other classes of pantothenate kinase may also be worth pursuing: Type II found in *Staphylococcus aureus* and Type III found in *Helicobater pylori* and *Pseudomonas aeruginosa* are not regulated by free CoAs and acyl-CoA [65, 66]. Overexpression of CoaA from *Pseudomonas putida* and the heterologous pathway for polyhydroxyalkanoate production in *E. coli* improved the polymer content 50% [67].

Alternatively, pyruvate accumulation could potentially be reduced by overexpression of genes in the pyruvate dehydrogenase complex, which converts pyruvate to acetyl-CoA [68]. This large complex is composed of three enzymes coded by the *aceE*, *aceF* and *lpd* genes. The production of poly(hydroxybutyrate), a product one enzymatic step beyond the 3-hydroxybutyryl-CoA intermediate of the current study (Fig. 1), has previously been shown to be improved 60% by the overexpression of the *aceEF* genes using a novel serine pathway [69]. Additional research overexpressing one or more component of this enzyme complex would determine whether an increased conversion of pyruvate to acetyl-CoA, coupled with citrate synthase variants which were the focus of this work, further improves formation of 3-HB and other products derived from acetyl-CoA.

### Competition between citrate synthase and the 3-HB pathway

During heterologous production of any biochemical, controlling the competition for the essential precursor between central carbon enzyme and the introduced pathway is critical to improve the biochemical production. Compared to the secondary or intermediate pathways, catalytic efficiency of primary central metabolism pathway such as glycolysis is generally high [70]. Therefore, when a heterologous pathway is introduced, the first enzyme in that pathway will compete directly for precursors with the efficient host enzyme. In this case, acetyl-CoA is the precursor for the introduced 3-HB pathway and competes with native citrate synthase. Under aerobic conditions, acetyl-CoA pools are reported to be 20–600 µM in *E. coli* cells [71]. Wild-type GltA has a K_M(acetyl−CoA)_ of 120–151 µM and a k_CAT_ of 34–81 s^− 1^ [17, 72, 73]. The first step of 3-HB production pathway in this study was catalyzed by overexpressed PhaA from *C. necator*, for which kinetic parameters with respect of acetyl-CoA were not found, though the kinetic parameters for the enzyme from Gram-negative *Zoogloea ramigera* are K_M(acetyl−CoA)_ of 390 µM and a k_CAT_ of 2.1 s^− 1^ [74, 75]. To estimate competition between the two enzymes by taking average kinetic values for GltA of K_M_ equal to 135 µM and a k_CAT_ of 50 s^− 1^, a maximum stated acetyl-CoA concentration of 600 µM, and applying Michaelis-Menten kinetics, the wild-type GltA will operate at 82% of its maximum rate (41 s^− 1^) while PhaA at will operate at 61% of its maximum (about 1.3 s^− 1^). Naturally this analysis provides only an estimate since the actual activity is impacted by numerous factors such as the presence of cofactors and complicated by regulatory networks. Nevertheless, the overexpression of PhaA would tend to decrease the concentration of acetyl-CoA, and at 200 µM, for example, the wild-type GltA will operate at 60% (30 s^− 1^) while PhaA will operate at 34% (0.1 s^− 1^). The ratio of rates between the two competing enzymes (rate of GltA/rate of PhaA) has therefore increased from 32 at 600 µM to 42 at 200 µM acetyl-CoA concentration. This estimation shows that increasing the overexpression of PhaA can be counterproductive as doing so decreases the effectiveness of the PhaA protein relative to the native GltA. One approach which is the focus of this work is to rebalance the competition between the native enzyme and the competing heterologous pathway by changing the kinetic parameters of the native enzyme. For example, the GltA[A267T] variant possesses kinetic parameters very similar to PhaA: a K_M(acetyl−CoA)_ of 351 µM and a k_CAT_ of 2.1 s^− 1^ [17]. At 600 µM acetyl-CoA, the ratio of activity between GltA and PhaA is 1.54, while at 200 µM acetyl-CoA, the ratio of activity between GltA and PhaA is essentially unchanged at 1.58. Thus, not only does altering kinetic parameters of native enzymes offer the prospect of allowing a heterologous pathway more effectively to compete kinetically with native pathways, but careful design of the modification on the native enzyme can create a system which is less sensitive to changes in intracellular concentrations which may occur with changes in nutrients or in operational mode.

### Nutrient limitation stategies

To increase the productivity and titer for 3-HB, strategies such as repeated batch, N-starvation/depleted/limited, and P-starvation/depleted/limited have been reported [1, 8, 19–21]. Compared to N-limitation, P-limited strategies provided greater 3-HB yield and productivity which was attributed to greater metabolic activity under P-limitation due to internal phosphate storage [19]. Previous research using phosphate-limited *B. subtilis* glucose cultures at 0.1 h^− 1^ demonstrated increased carbon flux towards the pentose phosphate pathway and transhydrogenase flux toward NADH compared to C-limited growth [76]. Because PhaB catalyzing acetoacetyl-CoA to hydroxybutyryl-CoA requires NADPH and is believed to be the rate-limiting step for 3-HB production [77], a phosphate depleted process was selected for the repeated batch using GltA[A267T]. In this study, this phosphate-starved repeated batch strategy produced a 15.9 g/L 3-HB (effective concentration of 21.4 g/L considering dilution effect of acid/base and glucose feed) with a yield of 0.16 g/g glucose (Fig. 7) without overexpressing additional genes such as *zwf* and *yciA/tesB/coaA* or supplementing glutamate. A low phosphate medium concentrations allowed the cultures to become phosphate depleted when the OD reached about 30, corresponding to the end of the first repeated batch. Despite deleting genes responsible for acetate formation, the 3.4 g/L acetate titer was observed, possibly due to promiscuous native thioesterase activity.

In conclusion, GltA variants in combination of process engineering offer an effective strategy to generate acetyl-CoA derived products by modulating carbon flow between TCA cycle for biomass production and an introduced pathway for the product formation. In case of 3-HB production, increasing K_M_ for acetyl-CoA or/and k_CAT_ of PhaA via protein engineering can divert greater acetyl-CoA towards 3-HB production. Pyruvate accumulation could be reduced by additional modifications to PDH to reduce inhibition from acetyl-CoA or/and NADH. In addition to protein engineering, chromosomal integration of 3-HB pathway under a constitutitve promoter will further improve the process by eliminating the need of antibiotic/inducer addition and reducing the metabolic burden to maintain the plasmid.

## Materials and methods

### Strains and genetic modifications

Strains used in this study are shown in Table 1, and plasmids are shown in Table S2. Gene knockout strains were constructed using lambda-red recombination [79] with primers having 50 bp homology (Table S3). Knockouts at gene location of *poxB*, *ldhA, pta-ackA*, and *gltA* were selected on Lysogeny Broth (LB) plates supplemented with kanamycin and confirmed using forward primers external to the target gene and reverse primers within the kanamycin resistance (Table S2). The kan^R^ marker was removed by expression of FLP recombinase from pCP20 [80], and confirmed by external primers to the gene target (Table S2).

A homologous recombination method was used to integrate point-mutated *gltA* variants in MEC1381. pKSI-gltA(W) plasmid (pHR-gltA) containing a point mutation in *gltA* was used as donor DNA for chromosomal integration. The point-mutated *gltA* variant, kanamycin cassette and 500 bp of flanking homology region were amplified from the respective plasmid and used to transform electrocompetent MEC1381 expressing the lambda red system from pKD46 [79]. Transformants were grown on LB plates supplemented with kanamycin [18]. The kan^R^ marker was removed, confirmed by PCR using external primers to the gene target (Table S2). Point-mutated *gltA* genes were amplified from the chromosome, gel purified, and sequenced to confirm correct mutations (ACGT, Inc., Wheeling, IL, USA).

### Plasmid construction

Plasmids were constructed using NEBuilder HiFi Assembly (New England Biolabs, Ipswich, MA, USA) or *Escherichia coli* DH5α-mediated assembly [81]. Phusion High-Fidelity Polymerase (New England Biolabs, Ipswich, MA, USA) or PrimeStar Max High-Fidelity Polymerase (Takara Bio, Mountain View, CA, USA) was used to amplify DNA for cloning and genome integration. Quick-DNA Miniprep and Zyppy Plasmid Miniprep Kits were used to purify genomic and plasmid DNA (Zymo Research, Irvine, CA, USA). DNA Clean and Concentrator and Zymoclean Gel DNA Recovery Kits were used to purify PCR fragments (Zymo Research, Irvine, CA, USA). Plasmids were confirmed by restriction digest (New England Biolabs, Ipswich, MA, USA) and sequencing (ACGT, Inc., Wheeling, IL, USA).

To construct pHR-gltA for sited-directed mutagenesis, a kanamycin cassette was amplified from pKD4 [79], the *gltA* gene from *E. coli* W with 570 bp of flanking DNA upstream and 584 bp downstream were cloned into the pKSI vector [82] using NEbuilder® Hifi assembly. The assembled plasmid was transformed into *E. coli* DH5α cells using chemical transformation. Point mutations were introduced to the plasmid using method described previously [18].

The genes *phaA* and *phaB* from *Cupriavidus necator* were codon optimized (Invitrogen, Thermo Fisher Scientific, Waltham, MA, USA) and cloned into the pTrc99A plasmid [83] to form plasmid pHR-AB. Each gene was constructed in an operon containing the trc promoter and the rrnB T1 terminator. The plasmid size was determined by restriction digest, and the sequence was confirmed by DNA sequencing method. The low copy plasmid pACYC184 [84, 85] containing the native thioesterase *yciA* (pHR-yciA), thioesterase *tesB* (pHR-tesB), or pantothenate kinase *coaA* (pHR-coaA) used Anderson promoter J23107 (http://parts.igem.org/Promoters/Catalog/Anderson; strength = 0.36), ribosome binding site RBS_B0034 (http://parts.igem.org/Part:BBa_B0034), and terminators identical to the native *E. coli* W genes. Each gene was cloned into the multiple cloning site of the vector using NEbuilder® Hifi assembly (NEB, Ipswich, MA, USA), chemically transformed into *E. coli* DH5α cells, and colonies isolated on plates with appropriate antibiotic. Each plasmid was confirmed by restriction digest and sequencing.

The plasmid pHR-AB was electroporated into all the *gltA* variant strains, the Δ*gltA* strain MEC1381, and the strain containing the wild-type *gltA* gene (MEC1365). The pHR-yciA, pHR-tesB, or pHR-coaA plasmid was transformed by electroporation into strains containing pHR-AB plasmid.

### Media and growth conditions

The strains were routinely cultured and maintained in Lysogeny Broth. For production of 3-HB, a defined medium to which glucose was added contained (per L): 8.0 g NH_4_Cl, 1.2 g KH_2_PO_4_, 1.0 K_2_HPO_4_, 2.0 g K_2_SO_4_, 0.6 g MgSO_4_·7H_2_O, 0.02 g thiamine HCl, 0.25 mg ZnSO_4_·7H_2_O, 0.125 mg CuCl_2_·2H_2_O, 1.25 mg MnSO_4_·H_2_O, 0.875 mg CoCl_2_·6H_2_O, 0.06 mg H_3_BO_3_, 0.25 mg Na_2_MoO_4_·2H_2_O, 5.5 mg FeSO_4_·7H_2_O, 50 mg citric acid, and 5.23 g (25 mM) or 31.35 g (150 mM) 3-[*N*-morpholino]propanesulfonic acid (MOPS) as indicated. Thiamine and trace metals solutions were filter sterilized, while other medium components were autoclaved in compatible mixtures and combined. Calcium pantothenate (final concentration 1 mM) was filter sterilized when used, as were antibiotics as required for plasmid maintenance or strain selection (final concentration): ampicillin (150 µg/mL), kanamycin (40 µg/mL), and chloramphenicol (30 µg/mL).

### Shake flask cultures

All shake flask studies were initiated by transferring a single colony from an LB plate to 3 mL LB liquid culture.

For growth rate studies of strains lacking plasmids, after 6–10 h of growth the LB culture was used to inoculate 3 mL defined medium containing 5 g/L glucose to an initial optical density (OD) of 0.05. When the OD reached 2–4, this culture was used to inoculate triplicate 250 mL baffled shake flasks containing 50 mL of defined medium with 5 g/L glucose in 150 mM MOPS to an initial OD of 0.02. Growth rate was determined by measuring the OD of 6–8 samples of each culture during an exponential growth [18].

For studies on 3-HB production using strains containing one or two plasmids, after 6–10 h of growth the LB culture was used to inoculate 3 mL defined medium containing 5 g/L glucose to an initial OD of 0.05. When the OD reached 2–4, 200 µL of this culture was used to inoculate triplicate 250 mL baffled shake flasks containing 20 mL of defined medium with 8 g/L glucose in 150 mM MOPS. Cultures of MEC1381 (the Δ*gltA* strain) were additionally supplemented with 1 g/L glutamate [25]. After growth to an OD of 0.4–0.6, cultures were induced with 200 µM IPTG (Isopropyl β-D-1-thiogalactopyranoside) [18], and after 6 h, samples were collected for the measurement of extracellular products. All shake flasks cultures were grown at 30 °C on a rotary shaker at 250 rpm.

### Batch and repeated batch processes

A single colony from an LB plate was used to inoculate 3 mL LB. After 6 h of growth, this culture was used to inoculate a 500 mL shake flask containing 50 mL of defined medium with 30 g/L glucose in 150 mM MOPS to an initial OD of 0.02. When the shake flask culture reached an OD of 1.5-2, the entire 50 mL contents were used to inoculate a 2.5 L bioreactor (Bioflo 2000, New Brunswick Scientific Co., New Brunswick, NJ, USA) containing 1.2 L defined medium with 30 g/L glucose in 25 mM MOPS. Cultures were induced with 50 µM IPTG at an OD of 0.6–0.8 [86]. Duplicate batch processes at 30 °C and 400 rpm were performed.

A repeated batch study was conducted with identical initial composition except 40 g/L glucose, 0.9 g/L MgSO_4_·7H_2_O and 10 g/L NH_4_Cl. The process was induced at an OD of 1 with 50 µM IPTG [84]. At OD of 18, 5 mM betaine was added as an osmoprotect [87]. Multiple times after the depletion of glucose, a 52 mL solution containing 39 g glucose and 50 mg ampicillin was introduced into the fermenter. This medium contains 14.6 mM P, and with a biomass yield on P of about 35 [88], becomes depleted in P when the OD reaches about 30.

In all bioreactor processes the dissolved oxygen was maintained above 40% of saturation by sparging air with oxygen as necessary at 1.25 L/min, with agitation at 400 rpm (batch) or 500 rpm (repeated batch). The pH was controlled using either 30% KOH (w/v) or 20% H_2_SO_4_ (w/v). Antifoam 204 (Sigma) was used as necessary [78].

### Analytical methods

The optical density at 600 nm (OD) (UV-650 spectrophotometer, Beckman Instruments, San Jose, CA, USA) was used to monitor cell growth. Samples were routinely frozen at -20 °C analysis, and thawed samples were centrifuged (13,000 × g for 5 min) and filtered (0.45 μm nylon filter, 13 mm, Agilent Technologies, CA, USA). High performance liquid chromatography using 4 mN H_2_SO_4_ at 60 °C and 0.6 mL/min with a Coregel 64-H ion-exclusion column (Transgenomic Ltd., Glasgow, United Kingdom), and refractive index detector was used to quantify glucose and organic acids [89]. Students T-test was used to compare data statistically, with 95% confidence interval the basis for significance.

### Electronic supplementary material

Below is the link to the electronic supplementary material.

Supplementary Material 1

## Data Availability

All processes data are available in the main text of the additional material. All raw data files and strains can be access by contacting the corresponding author.
