# Supplementary material for: Citrate synthase variants improve yield of acetyl-CoA derived 3-hydroxybutyrate in Escherichia coli
Source: Microb Cell Fact. 2024 Jun 12;23:173. doi: 10.1186/s12934-024-02444-8 (PMC11167817; doi:10.1186/s12934-024-02444-8)
Supplement: Supplementary file 1 — Supplementary Material 1 [file 12934_2024_2444_MOESM1_ESM.pdf]

Supplemental Material

Citrate synthase variants improve yield of acetyl-CoA derived 3-hydroxybutyrate in *Escherichia coli*

Hemshikha Rajpurohit, Mark A. Eiteman

Table S1. Concentrations of biochemical products accumulated after 6 h in shake flask studies using glucose as the sole carbon source (Δ*gltA* strain also contained nominally 2 g/L glutamate). All strains contained knockouts of the *pta-ackA*, *poxB* and *ldhA* genes. These are the raw data from which yields were calculated in the referenced figures.

Citrate synthase variant	Overexpressed Genes	Glucose initially (g/L)	Glucose at 6 h (g/L)	3HB at 6h (mg/L)	Acetate at 6 h (mg/L)	Pyruvate at 6 h (mg/L)	Ref.
Wild-type	*phaA phaB*	7.69	0.0	142	146	44	Fig. 2
K167A	*phaA phaB*	8.68	2.50	604	92	1150	Fig. 2
A267T	*phaA phaB*	7.69	5.38	233	52	470	Fig. 2
V361A	*phaA phaB*	8.68	2.10	631	99	1164	Fig. 2
M372S	*phaA phaB*	10.62	5.64	201	14	0	Fig. 2
F383M	*phaA phaB*	8.68	2.70	613	92	1163	Fig. 2
Δ*gltA*	*phaA phaB*	7.98	2.76	647	120	1252	Fig. 2
Wild-type	*phaA phaB tesB*	7.81	0.75	351	269	41	Fig. 3
K167A	*phaA phaB tesB*	7.96	6.30	258	170	8	Fig. 3
A267T	*phaA phaB tesB*	7.81	4.87	389	254	20	Fig. 3
V361A	*phaA phaB tesB*	7.81	3.95	514	316	29	Fig. 3
M372S	*phaA phaB tesB*	6.61	2.73	165	108	14	Fig. 3
F383M	*phaA phaB tesB*	7.96	5.58	332	250	8	Fig. 3
Δ*gltA*	*phaA phaB tesB*	7.96	3.96	769	375	54	Fig. 3
Wild-type	*phaA phaB yciA*	8.50	2.16	141	897	80	Fig. 4
K167A	*phaA phaB yciA*	8.50	5.76	119	743	38	Fig. 4
A267T	*phaA phaB yciA*	8.32	6.16	65	392	12	Fig. 4
V361A	*phaA phaB yciA*	8.50	5.14	142	822	23	Fig. 4
M372S	*phaA phaB yciA*	6.61	1.54	121	637	13	Fig. 4
F383M	*phaA phaB yciA*	8.50	6.61	80	542	25	Fig. 4
Δ*gltA*	*phaA phaB yciA*	8.50	4.52	120	1379	0	Fig. 4
Wild-type	*phaA phaB coaA*	8.63	0.37	0	41	0	Fig. 5
K167A	*phaA phaB coaA*	8.63	6.82	197	33	118	Fig. 5
A267T	*phaA phaB coaA*	8.63	7.23	152	28	169	Fig. 5
V361A	*phaA phaB coaA*	8.63	5.86	298	51	115	Fig. 5
M372S	*phaA phaB coaA*	6.25	0.21	109	0	13	Fig. 5
F383M	*phaA phaB coaA*	8.63	6.00	287	44	134	Fig. 5
Δ*gltA*	*phaA phaB coaA*	8.63	5.03	441	89	352	Fig. 5

Table S2. Plasmids used in this study.

Name	Relevant characteristics	Description	Reference/Source
pKD4	Amp^R, Kan^R; R6K ori	Source of Kan^R cassette	[1]
pKD46	Amp^R; pSC101 ori (ts); *araBAD* promoter for λ-Red genes	λ-Red helper plasmid	[1]
pCP20	Amp^R, Cam^R; pSC101 ori (ts)	Expression of FLP recombinase	[2]
pTrc99A	Amp^R; pBR322 ori	IPTG inducible expression, trc promoter	[3]
pACYC184	Cam^R, Tet^R; p15A ori	Low copy number plasmid	ATCC Product-37033™
pKSI-I	Amp^R; Kan^R; pUC ori	pBluescript II KS(-) backbone with I-SceI site–MCS–I-SceI site cassette	[4]
pHR-gltA	Amp^R; pUC ori	pKSI-I + *gltA* + Kan^R	This study
pHR-AB	Amp^R; pBR322 ori	pTrc99A::trc-*phaA*-rrnB1, trc-*phaB*-rrnB1	This study
pHR-tesB	Cam^R ; p15A ori	pACYC184::J23107- RBS_B0034-*tesB*-*tesB* terminator	This study
pHR-yciA	Cam^R ; p15A ori	pACYC184::J23107- RBS_B0034-*yciA*-*yciA* terminator	This study
pHR-coaA	Cam^R ; p15A ori	pACYC184::J23107- RBS_B0034-*coaA*-*coaA* terminator	This study

Table S3. Primers used in this study.

Name	Description	Sequence 5'-3'
MEP144	gltA_F	TACGCATGGGATATGAGGCGGTACAG
MEP145	gltA_R	GTGACTGACGAATCACCACGTTATCACC
MEP310	Up-gltA-F	TCATGCAAAACACTGCTTCCAGATG
MEP330	Seq-F	CTTTCCGCCTGATGGGCTTCG
MEP417	M13R	CAGGAAACAGCTATGAC
MEP477	pKSI-HA-gltA-R	CTTGATATCGAATTCCTGCAGTCATGCAAAACACTGCTTC
MEP505	pksUps_HACS	TGCTTTTGTATCAGCCATTTAAGGTCTCCTTAGCGCC
MEP508	CSend_HAKan	GGACCATGGCTAATTCCCATTAACGCTTGATATCGCT
MEP509	Kan_HApksDwn	ACAACTTAGCAATCAACCAGTGTAGGCTGGAGCTGCTTC
MEP510	Kan_HACSend	AGCGATATCAAGCGTTAATGGGAATTAGCCATGGTCC
MEP698	phaA_fwd	GCTCGGTACCCGGGGATGACCGATGTTGTTATTGTTAG
MEP699	phaA_rev	TCGACTCTAGAGGATTTATTTACGTTCAACTGCCAG
MEP700	pTrc99A_fwd	GTTGAACGTAAATAAATCCTCTAGAGTCGACCTGCAG
MEP701	pTrc99A_rev	AACAACATCGGTCATCCCCGGGTACCGAGCTCG
MEP714	phaB_fwd	GCTCGGTACCCGGGGATGACCCAGCGTATTGCATATG
MEP715	phaB_rev	TCGACTCTAGAGGATTTAACCCATATGCAGACCAC
MEP716	pTrc99A_fwd	CTGCATATGGGTTAAATCCTCTAGAGTCGACCTGCAG
MEP717	pTrc99A_rev	AATACGCTGGGTCATCCCCGGGTACCGAGCTCG
MEP735	phaA_5'	ATGACCGATGTTGTTATTGTTAGCGC
MEP736	phaA_3'	TTATTTACGTTCAACTGCCAGGGC
MEP737	phaB_5'	ATGACCCAGCGTATTGCATATGTTAC
MEP738	phaB_3'	TTAACCCATATGCAGACCACCAT
MEP739	ptrc99A_phaA_fwd	GGCGGGCAGGACGCCCGCCCGCCATAAACTGCC
MEP740	ptrc99A_phaA_rev	CGCAAAAAACATTATAATCCGCTCCCGGCGGAT
MEP741	phaB_fwd	CGCCGGGAGCGGATTATAATGTTTTTTGCGCCGACATCATAACGGTTCTGGC
MEP742	phaB_rev	AGTTTATGGCGGGCGGGCGTCCTGCCCGCCACC

MEP827	MEP329_F	CCAGTGCGGCCAATGTTTTTTGT
MEP 828	MEP310_F	ACTGATTAATGTGGCTGGTGG
MEP1070	KS (gltA-W)-fwd	CCACCAGCCACATTAATCACCGGGGGATCCACTAGTTCTAG
MEP1071	DS (gltA-W)- rev	CTAGAACTAGTGGATCCCCCGGTGATTAATGTGGCTGGTGG
MEP1145	pACYC184-F	GAGTCAACGCCATGAGCGGCCTCATTTCTTATTCTGAGTT
MEP1146	pACYC184-R	CTTTCACTAGTAATAATACCTAGGGCTGAGCTAGCCGTAAATCAGTCATAGTATCGTGG
MEP1147	tesB-(pACYC184)-F	CCTAGGTATTATTACTAGTGAAAGAGGAGAAATACTACATGAGTCAGGCGCTCAAA
MEP1148	tesB-(pACYC184)-R	GCCGCTCATGGCGTTGACTCGGCGAGAATGCCTATAACGC
MEP1149	yciA (pACYC184)-F	CCTAGGTATTATTACTAGTGAAAGAGGAGAAATACTACATGTCTACAACACATAACGT
MEP1150	yciA (pACYC184)-R	GGCCGCTCATGGCGTTGACTCTAACGGCACCACCGAAATT
MEP1151	pACYC184-gene-seq F	GGTTTCCGTGTTTCGTAAAGTC
MEP1152	pACYC184-gene-seq R	GCGGACTGTTGTAACTCAGA
MEP1153	pTrc99A-phaA-seq R	GGATCACCTTTACGCTGCGG
MEP1154	pTrc99A-phaB-seq R	CCTCTTGTGCCAGTGCCATG
MEP1155	pTrc99A-AMP-seq R	GGCGACACGGAAATGTTGAA
MEP1162	coaA-pACYC184_ F	GGTATTATTACTAGTGAAAGAGGAGAAATACTACATGACGCCTTACCTACAGTTTG
MEP1163	coaA-pACYC184_ R	GGCCGCTCATGGCGTTGACTCTGAAATATCCCTGCGTAGTGC

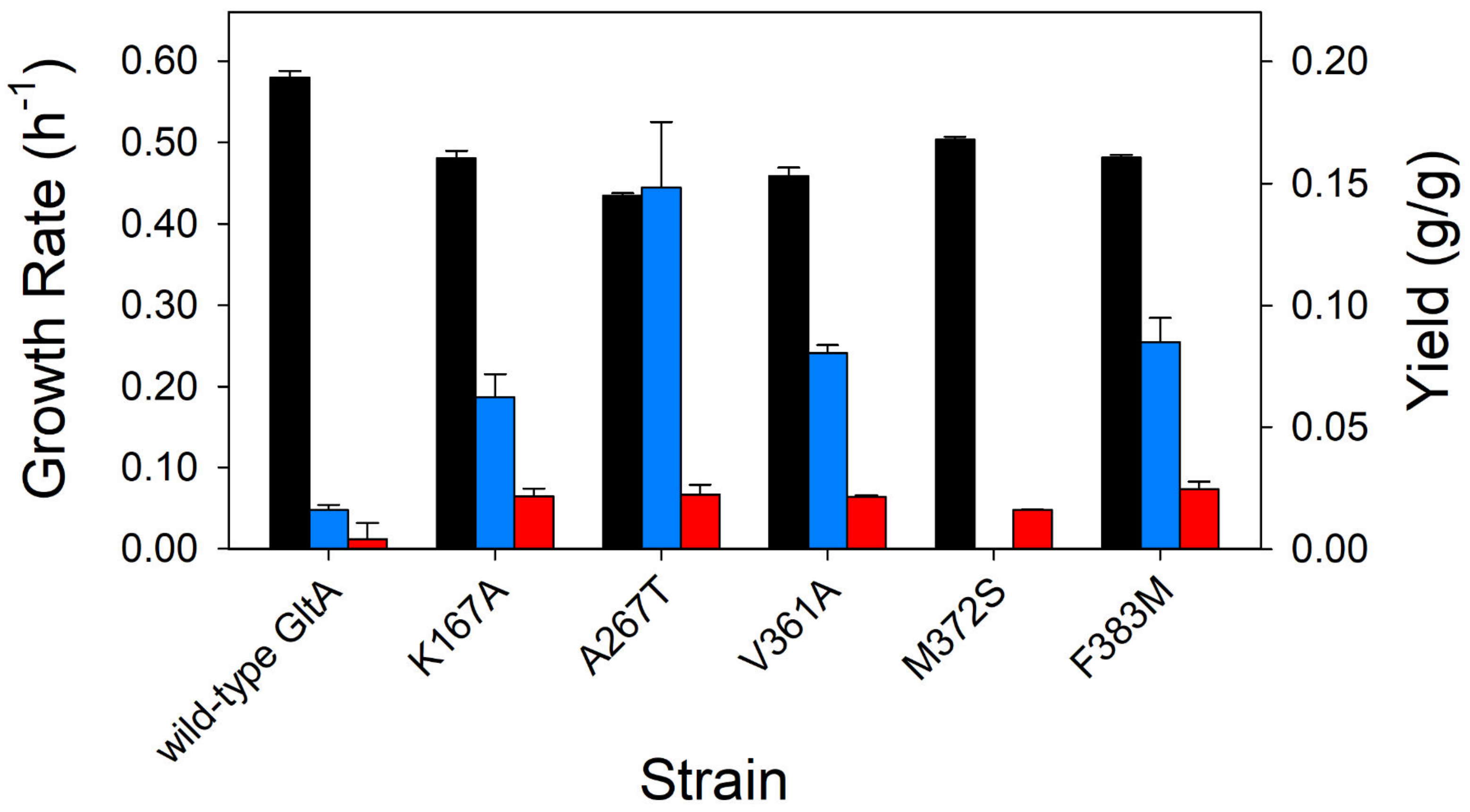

Figure S1. Growth rate (h^{-1}) (black), yield of pyruvate (blue) and acetate (red) from glucose in citrate synthase variants at 30°C. All strains have deletions in *poxB*, *ldhA*, *pta* and *ackA* genes. MEC1365 expresses wild-type GltA, and MEC1381 has a *gltA* gene knockout.

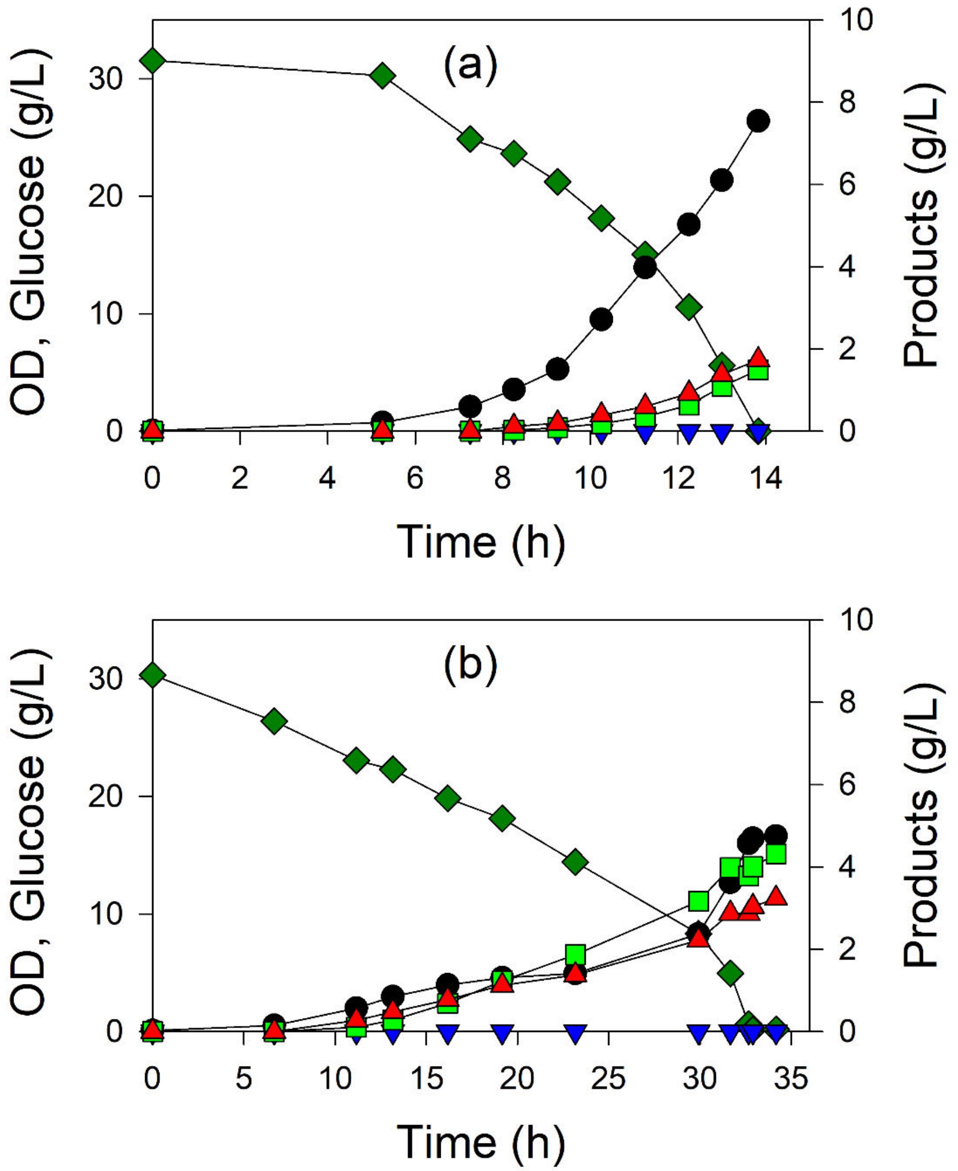

Figure S2. Controlled growth of W Δ*ldhA* Δ*poxB* Δ*pta-ackA* strains expressing different chromosomal *gltA* genes coding citrate synthase enzymes in defined medium with 30 g/L glucose and 25 mM MOPS, induced with 50 μM IPTG. Each strain expressed genes in the 3-HB pathway via plasmid pHR-AB as well as TesB via plasmid pHR-tesB. Concentrations of glucose (◆), pyruvate (▼), OD (●), acetate (▲), 3-HB (■) were measured during the course of the 1.25 L batch processes. (a) wild-type GltA, (b) GltA[K167A].

References

1. Datsenko, K. A., Wanner, B. L. (2000). One-step inactivation of chromosomal genes in *Escherichia coli* K-12 using PCR products. *Proc Nat Acad Sci USA* 97(12), 6640–6645.
2. Cherepanov, P.P., Wackernagel, W. (1995). Gene disruption in *Escherichia coli*: Tc^R and Km^R cassettes with the option of Flp-catalyzed excision of the antibiotic-resistance determinant. *Gene* 158, 9-14.
3. Amann, E., Ochs, B., Abel, K.-J. (1988). Tightly regulated *tac* promoter vectors useful for the expression of unfused and fused proteins in *Escherichia coli*. *Gene* 69, 301-315.
4. Yang, J., Sun, B., Huang, H., Jiang, Y., Diao, L., Chen, B., Xu, C., Wang, X., Liu, J., Jiang, W. (2014). High-efficiency scarless genetic modification in *Escherichia coli* by using lambda red recombination and I-SceI cleavage. *Appl Environ Microbiol* 80:3826-3834.
